# Supplementary material for: Predictive significance of joint plasma fibrinogen and urinary alpha-1 microglobulin-creatinine ratio in patients with diabetic kidney disease
Source: PLoS One. 2022 Jul 8;17(7):e0271181. doi: 10.1371/journal.pone.0271181 (PMC9269903; doi:10.1371/journal.pone.0271181)
Supplement: Attachment — Submitted filename: Response to reviewers.docx [file pone.0271181.s001.docx]

**Reply to the reviewers’ comments**

**Reviewer #1:**

1. The introduction section is not strong, it is poorly written. For this clinical study, a much better introduction should be provided. the importance of early diagnosis of DKD must be emphaszied.

**Reply:** We added more contents in line 45-55 in **Introduction** section.

2. The study must present a hypothesis toward the end of the introduction section.

**Reply:** we have added a hypothesis at the end of the introduction section**.**

3. I partially agree with the conclusions. While the authors stated that joint plasma FIB concentration and urinary α1-MG/CRratio can be used as a powerful predictor for general and advanced DKD. Looking at the AUC, I believe these values are good for general DKD only, not for advanced disease.

**Reply:** we have corrected the related discussion in the 2^nd^, 3^rd^ and 4^th^ paragraphs and the conclusion.

4. How many of the DKD patients were advanced, non-advanced and how many did not have DKD?

**Reply:** The advanced DKD and non-DKD patients have been defined as stage II+III and stage IV, respectively, in **Patients population** section. The amounts of advanced DKD and non-DKD were 36 and 81, respectively, which was descripted in **Basic characteristics and biomarkers of pure T2D and DKD patients** section in the original manuscript. We have revised the description in line 137-138.

5. Discussion of this article can be improved by emphasising on the current knowledge and the potential of novel markers and their impact on healthcare.

**Reply:** We added some discussion in the second, third and fourth paragraphs of the Discussion section.

**Reviewer #2:**

1. Predictive value of plasma fibrinogen in DKD is not a novel finding (ref.12), and urinary alpha1 microglobulin is also an established biomarker of non-specific tubular injury. Therefore, the authors should, as the next step, study a prospective cohort to validate the significance of fibrinogen, for example.

**Reply:** Our study was a retrospective study, therefore, we aimed to further carry out a prospective study to validate the conclusion. In **Conclusions** section, we refreshed the statement.

2. Specificity of these markers to DKD should also be investigated by studying non-diabetic CKD patients as a control group.

**Reply:** In this study, we did not include non-diabetic CKD, thus, based on the present data, we can not study its specificity. We may include these patients as a control group in further study to draw a more definitive conclusion.

3. The additional value of combining FIB and a1-MG/CR is also marginal when compared with a1-MG/CR alone, and it is difficult to find advantage of combining these two markers.

**Reply:** Although a1-MG/CR is a sensitive diagnostic biomarker of tubular injury, and it also exhibited a high identifying value for DKD with an AUC of 0.972, which was similar to that of combining FIB and a1-MG/CR, the combination showed a much higher predictive value than that of a1-MG/CR single use. These results were consistent with the major aim of our study in assessing the predictive significance.

**Reviewer #3:**

The strength of this study is the confirmation of DKD by renal puncture pathology. However, there are some major concerns to be addressed to support the results and conclusion.

1. The authors divided 117 DKD patients into two groups including test group (59 subjects) and validation group (58 subjects). What is the standard of grouping? Random or others? Is there the possibility of selection bias?

**Reply:** We grouped the DKD patients according to the 1:1 principle in random. The grouping method was as follows: we first numbered the patients from No. 1 to 117, then selected 117 numbers from the random number table to assign a random number to each patient. Subsequently, we ranked the 117 random numbers in ascending order, and selected the first 59 cases as the test group, and the last 58 ones as the validation group. Finally, we compared the differences of the variables between the two groups, no significant differences were found (p>0.05 for all), which has been described in **Correlation analysis of plasma FIB with urinary proteins** section.

2. Is there DKD patients with normoalbuminuria but elevated SCr levels? What is the result of joint plasma FIB concentration and urinary α1-MG/CR ratio in these patients?

**Reply:** In our original data, there was 6 DKD patients with normoalbuminuria (<3.0 mg/mmoL), but only one patient (male) exhibited elevated sCR level (251.2µmol/L, compared with the median 86.8µmol/L in all T2D patients and the upper limit 133µmol/L of reference interval for males). However, except for the 2 missing results of the combination in the 6 DKD patients, the levels of the rest four were 0.50-1.0, and all were beyond the median of 0.47 in T2D patients.

3. The statistical results indicated that joint FIB and α1-MG/CR have a low power in identifying patients with advanced DKD from non-advanced DKD. However, when performing regression analysis by treating FIB and α1-MG/CR as categorical variables based on the optimal cut-off values (5.58 g/L and 11.07 mg/mmol, respectively) from ROC curve analysis, joint FIB and α1-MG/CR demonstrated a high OR value (3.252) in prediction for advanced DKD. How does the author explain this result？

**Reply:** Within our knowledge, we think that the ROC analysis can indicate the diagnostic power, but the regression analysis can reveal the predictive power. Therefore, joint FIB and α1-MG/CR may be have a relatively lower identifying value and a higher predictive significance for advanced DKD. In fact, the result of univariate analysis of the combination did not show an OR with statistical difference, and the levels also exhibited no statistical differenc between the three stages. Based on these results, we revised the text in the fourth paragraph of the **Discussion** section and in **Conclusion** section.

4. In this study, there are some parameters with missing data, the author should present the proportion and processing of missing data.

**Reply:** We presented the percent of missing data and the processing method in ;ine 144-148 of the **Basic characteristics and biomarkers of pure T2D and DKD patients** section.

5. There are typos or spelling mistakes, such as the “parametes” at line 135, page 7. The authors should go through the whole manuscript and make revisions.

**Reply:** We corrected the mistakes we could found.
